# Prevalence of Mental Health Disorders and Their Associated Risk Factors Among People Living with HIV in Rwanda: A Cross-Sectional Study

**DOI:** 10.1007/s10461-024-04358-3

**Published:** 2024-05-12

**Authors:** Laura Risbjerg Omann, Valentine Dushimiyimana, Emmanuel Musoni-Rwililiza, Caroline Juhl Arnbjerg, Vivianne Umuhire Niyonkuru, Jean Damascene Iyamuremye, Michel Gasana, Jessica Carlsson, Per Kallestrup, Christian Kraef

**Affiliations:** 1 Department of Public Health, Center for Global Health, Aarhus University, Aarhus, Denmark; 2 Rwanda Biomedical Centre (RBC), Kigali, Rwanda; 3 Ghent University, Ghent, Belgium; 4 College of Medicine and Health Sciences University of Rwanda, Kigali, Rwanda; 5 Mental Health Department, University Teaching Hospital of Kigali, Kigali, Rwanda; 6 Competence Centre for Transcultural Psychiatry, Mental Health Centre Ballerup, Copenhagen University Hospital–Mental Health Services CPH, Copenhagen, Denmark; 7 Department of Clinical Medicine, University of Copenhagen, Copenhagen, Denmark; 8 Research Unit for General Practice, Aarhus, Denmark; 9 Department of Infectious Diseases, Rigshospitalet, Copenhagen, Denmark; 10 Centre of Excellence for Health, Immunity and Infections (CHIP), Rigshospitalet, University of Copenhagen, Copenhagen, Denmark; 11 Heidelberg Institute of Global Health, Heidelberg University, Heidelberg, Germany

**Keywords:** Mental health, People living with HIV, Global health, Sub-Saharan Africa, Low- and middle-income countries

## Abstract

While life expectancy of people living with HIV is increasing, their burden of non-communicable diseases, including mental health disorders, is growing as well. The aim of this study is to investigate the prevalence and identify the risk factors associated with mental health disorders among this population in Rwanda. This cross-sectional study enrolled people living with HIV from 12 HIV clinics across Rwanda using random sampling. Trained HIV nurses conducted the Mini International Neuropsychiatric Interview to estimate the prevalence of major depressive episode, post-traumatic stress disorder, and generalized anxiety disorder. Sociodemographic, psychosocial, and HIV-related data were also collected. Associated risk factors for being diagnosed with one of the mental health disorders were assessed using modified Poisson regression with robust error variance. Of 428 participants, 70 (16.4%) had at least one mental health disorder with major depressive episode being most prevalent (n = 60, 14.0%). Almost all participants were adherent to antiretroviral therapy (n = 424, 99.1%) and virally suppressed (n = 412, 96.9%). Of those diagnosed with a mental health disorder, only few were aware of (n = 4, 5.7%) or under treatment for this mental health disorder (n = 5, 7.2%). Mental health disorders were associated with experiences of HIV-related stigma and discrimination (aRR = 2.14, 95%CI 1.30–3.53, p = 0.003). The results demonstrate underdiagnosis and undertreatment of mental health disorders among Rwandan People Living with HIV. Using HIV nurses to diagnose mental health disorders could serve as a low-cost strategy for integrating mental health care with existing HIV services and could inspire the implementation in other low-resource settings.

**Supplementary Information:**

The online version contains supplementary material available at 10.1007/s10461-024-04358-3.

## Background

Globally, more than 38.4 million people are living with HIV [1], and the majority is living in low- and middle-income countries (LMICs). While antiretroviral therapy (ART) has reduced the number of HIV cases progressing to AIDS [2, 3], non-communicable diseases (NCDs) are an increasing cause of morbidity and excess mortality among people living with HIV (PLWH) [4–6]. Mental health disorders are among the NCDs which pose a substantial burden of disease for this population [7–10]. According to a recent systematic review, up to 62% of PLWH experience mental health symptoms [10], and particularly depression is common among PLWH with prevalence rates up to 4 times higher than in the general population [10–14]. In addition to the burden of disease, comorbidity of mental health disorders among PLWH can complicate medication adherence [15–17], jeopardize viral suppression [18, 19], and increase the risk of clinical HIV progression [20, 21].

High prevalence rates of mental health disorders among PLWH have been reported in sub-Saharan Africa [22–24], where more than half of all PLWH reside [25]. While HIV health care services have been successfully implemented in many high-income countries, mental health services remain inadequate in many LMICs, exacerbated by a lack of specialists and varying degrees of mental health literacy [26–28]. This directly challenges the 95–95–95 goals defined by UNAIDS; 95% of PLWH should know their status, 95% of PLWH who know their status should be on ART treatment, and 95% of PLWH on treatment should be virally suppressed by 2025 [29, 30]. The burden of mental health disorders experienced by PLWH can partly be explained by several factors such as discrimination [31, 32] and HIV-related inflammation [33–35]. Furthermore, some studies suggest that certain ART drugs such as efavirenz may play a role in developing depression, but without uniform evidence [36–38]. Some severe mental health disorders e.g., schizophrenia, bipolar disorder, and psychosis may also be associated with sexual risk behaviour [39–41].

In the East African country Rwanda, 230,000 people are living with HIV [42]. During the genocide against the Tutsi in 1994, hundreds of thousands were victims of sexual violence, and HIV and AIDS were used as weapons of war, increasing the prevalence of HIV in the country [43, 44]. The population-wide Rwanda Mental Health Survey (RMHS) in 2018 found a total population prevalence of mental disorders of 23%, but utilization of mental health services in the general population remains low [45]. Also, knowledge about the prevalence of mental health disorders among PLWH in Rwanda is sparse [46, 47], and more research on the integration of mental health and HIV care from LMIC contexts such as Rwanda is needed [48].

Due to this research gap on prevalence and risk factors for mental health disorders among PLWH in Rwanda and other LMICs, the overall objectives of this study were to assess the prevalence of major depressive episode (MDE), post-traumatic stress disorder (PTSD), and generalized anxiety disorder (GAD) in a cross-sectional sample of PLWH in Rwanda using the validated Kinyarwanda version of the Mini International Neuropsychiatric Interview (MINI) [45, 49] and to assess differences in the prevalence compared to the background population of Rwanda as found in the RMHS [45]. Furthermore, the study aimed to explore the association between mental health disorders and HIV outcomes including ART adherence and viral suppression and examined psychosocial and sociodemographic risk factors for these mental health disorders.

## Methods

### Study Design

This study is a cross-sectional study assessing the prevalence of mental health disorders in a cohort of PLWH in Rwanda.

### Setting

The study was conducted in Rwanda from September to December 2022. Twelve health facilities representing all provinces of Rwanda were selected as the data collection sites to ensure representativeness. See Table 3—supplementary for further details of the sites and their geographical location.

### Study Size

The sample size was determined based on the prevalence of MDE among the background population in Rwanda, which has been estimated at 12% in the RMHS from 2018 [45]. The aim was to detect a prevalence of MDE among PLWH of 17% corresponding to an absolute difference of 5% compared to the general population. Setting the power at 80% and using a two-sided significance level of 5%, the calculated sample size was 364 participants. After accounting for 20% of non-responders, a sample size of 437 participants was decided.

### Participants

**Fig. 1:**
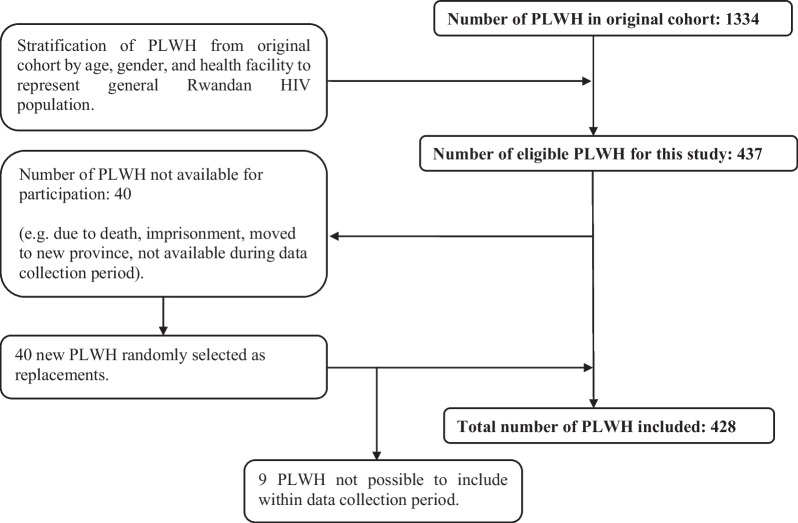
Flowchart of the inclusion process. The original cohort consisted of 1334 PLWH in Rwanda. These were stratified by age, gender, and health facility to ensure representativeness of the general Rwandan HIV population. Afterwards, 437 PLWH were included in this study. Of these, 40 PLWH were not available for participation, and therefore 40 PLWH were selected as replacements. However, 9 PLWH were not possible to include during the data collection period. Thus, the total sample size was 428 PLWH

The participants were enrolled from an existing cohort of 1334 PLWH in Rwanda. The original cohort consisted of PLWH ≥ 18 years old, with valid health insurance, without major NCDs, committed to long-term follow-up, not pregnant or < 3 months post-partum, and who were able to sign informed consent. Major NCDs were defined as self-reported cardiovascular disease, diabetes, cancer, or clinically confirmed hypertension, which was assessed at the HIV clinics before enrolment. HIV nurses at the 12 health facilities were contacted and informed about the inclusion criteria after which the clinic registers were cross-checked for all PLWH fulfilling the criteria. After identifying eligible PLWH, these were stratified by gender and age and selected by proportionate random sampling to represent the general HIV population of Rwanda. The number of PLWH needed to be included per site for the study was decided proportionately to the total number of PLWH affiliated with each site. Afterwards, the PLWH were included based on an age and gender distribution similar to that of the PLWH affiliated with the specific site. As this study aimed to include 437 of the 1334 PLWH in the original cohort, the number needed to be included from each site was reduced by approximately a factor three, still following the same age and gender distribution. The inclusion process of this study is further described in Fig. 1. Eligible PLWH were contacted, informed about the study, and enrolled if willing to sign informed consent. To compensate for transport costs, each participant was paid 5000 RWF (ca. 4.6 USD) on the day of the appointment. If a participant was unable to participate, another participant with the same gender and age group was selected randomly.

### Data Collection

Data were collected by trained HIV nurses at the selected health facilities using Research Electronic Data Capture (REDCap). Before participating in data collection, the data collectors completed a 3-day educational workshop in conducting the Mini International Neuropsychiatric Interview and collecting the data in REDCap, where they received detailed training on mental health and data collection by a Rwandan psychiatrist. The workshop included information on the structure of the interview instrument, the MINI, and roleplay to practice the interviews. During the data collection period, the nurses were frequently supervised on site by the principal investigator and mental health workers from Rwanda Biomedical Centre. Also, back-up supervision from a distance was ensured by almost daily calls or written contact between members of the research team and the data collectors. Moreover, the research team continuously supervised the data collection in REDCap.

### Outcomes

To diagnose the main outcomes MDE, PTSD, and GAD, the MINI was used in the local language Kinyarwanda. The MINI has previously been validated in a primary care context [50], and the Kinyarwanda version of the MINI was validated in Rwanda in connection to the RMHS [45] with translation into Kinyarwanda and subsequent reverse translation into English. The translation process was reviewed by a team of researchers and mental health experts [49]. The MINI diagnosed MDE based on current (last two weeks), past (more than two weeks ago), and recurrent (both current and past) episodes with an episode characterized by the presence of symptoms that persist for at least two weeks. In this study, the primary focus centered on determining whether participants had been diagnosed with MDE or not irrespective of whether the episode was current, past, or recurrent, aligning with the methodological approach employed in the RMHS to enable comparison of the MDE prevalence. PTSD was diagnosed based on symptoms during the past month, while GAD was based on symptoms during the past 6 months.

### Exposures

To explore associations with the participant’s sociodemographic data, data on age, gender, civil status, educational level, occupation, monthly income, and socioeconomic status were collected by interview.

The socioeconomic status of Rwandan citizens is defined by the Ubudehe categorization. In this study, the 2015 version of the Ubudehe categorization was used which stratifies the population into category 1 (lowest socioeconomic status; very poor citizens e.g. facing difficulties to have food) to 4 (highest socioeconomic status; e.g. chief executive officers of big businesses) [51].

Questions on family history of mental illness, self-reported symptoms or diagnoses of mental health disorders before and after HIV diagnosis, and experiences of HIV stigma and discrimination were also included to assess the participant’s current and previous mental health status. HIV-related data on time since HIV diagnosis and ART initiation, type of ART, viral load from last and previous visits, CD4 count at time of HIV diagnosis, and ART adherence was collected from patient files and interviews. Viral suppression was defined as HIV-RNA viral load < 200 copies/mL.

In Rwanda, HIV management is based on a person-centered approach using the differentiated model for ART service delivery (DSDM) [52, 53]. PLWH are stratified into the categories stable A1 (from 2022 defined as stable A) (adults on first-line or second-line ART for 6 months with one recent viral load < 200 copies/mL and good adherence), A2 (from 2022 defined as stable B) (adults eligible for stable A1, but considered likely to non-adhere, children > 2 years, adolescents (15–24 years), key population, clients with controlled comorbidities e.g. NCDs, mental health problems, and undernutrition, pregnant and breastfeeding women, clients shifted from unstable category), B (from 2022 defined as stable C) (disabled PLWH or in palliative care, unattended children, adults > 65 years), and unstable (clients with unsuppressed viral load > 200 copies/mL, advanced HIV disease (CD4 < 200), on third-line ART, less than 6 months on ART, with acute malnutrition, or unstable mental disorders). This model aims to reduce the frequency of HIV clinical and pharmacy visits for the well-controlled PLWH. Thus, for PLWH in group stable A1 and A2, the HIV clinical visits are reduced to every 6 months with pharmacy visits every 6 months for stable A1 clients and every 3 months for stable A2 clients. For clients belonging to the category stable B or unstable, the clinical visits take place every 3 months, while pharmacy visits are required every 3 months or monthly [53]. Data on the DSDM category of each participant was also collected.

### Statistical Methods

Descriptive analysis of the participants’ sociodemographic, psychosocial, and HIV-related data was presented as frequencies (absolute and relative) for categorical variables. Continuous variables were checked for normality using quantile–quantile plots and histograms. For normally distributed continuous variables, means with standard deviation (SD) were presented, while medians with interquartile range (IQR) were presented for continuous variables not following a normal distribution.

The prevalence of MDE and PTSD among PLWH estimated in this study were compared with the results of the RMHS from 2018 [45] using Pearson’s chi-squared test. Eligible participants for the RMHS were Rwandan citizens aged 14–65 years living in Rwanda at the time of the survey and who had been living in their respective areas for at least 6 months. Citizens with limited communication abilities preventing the completion of the interview were excluded.

Using a modified Poisson regression approach with robust error variance [54], bivariate analysis was performed, and risk ratios (RR) and corresponding 95% confidence intervals (CIs) were calculated for the associations between having either any mental health disorder, MDE, PTSD, or both and selected sociodemographic, psychosocial, and HIV-related data. Afterwards, a multivariable model based on the same Poisson regression was used to estimate the adjusted RRs (aRR) in order to examine the isolated associations between each of these variables and the mental health disorders. The variables age group, gender, socioeconomic class, DSDM category, family history of mental illness, self-reported mental health symptoms or diagnosis before HIV diagnosis, if HIV status had negative impact on social relations or sexual life, and HIV-related stigma and discrimination were included in the multivariable model. Of these variables, age group was selected a priori, while the other variables were included if they were found to be significant in the bivariate model (p < 0.05). Multi-collinearity was assessed using the Variance Inflation Factor (VIF). As all of the VIFs were ≤ 5, no significant multi-collinearity was present. A complete-case analysis was used due to the very low levels (< 3%) of missingness for each of the variables included in the multivariable model. Statistical analysis was performed in R version 4.2.2 for MacOS. Ethical approval was obtained from Rwanda National Ethics Committee.

## Results

### Characteristics of Participants

Four hundred and twenty-eight PLWH were included in the study of which nearly two-thirds were female (n = 270, 63.1%), and the mean age was 44.1 years (SD 11.5). More than half of the participants (n = 221, 52.0%) had completed primary school, while only 12.2% (n = 52) had obtained higher educational levels. Socioeconomic category (Ubudehe) 2 was most prevalent (n = 194, 45.3%). Table 1 illustrates the sociodemographic, HIV, and psychosocial characteristics of the study participants grouped according to mental health diagnosis.

**Table 1:** Sociodemographic, HIV, and psychosocial characteristics of participants by diagnostic group

		All (N = 428)^a^	No diagnosis (n = 358)	Any diagnosis (n = 70)	MDE only (n = 46)	PTSD only (n = 9)	MDE and PTSD (n = 14)
Sociodemographic data							
Age (years), mean (SD)		44.1 (11.5)	44.2 (11.4)	43.6 (11.9)	40.6 (10.4)	50.7 (12.1)	48.6 (14.0)
Age group, n (%)	≤ 24	22 (5.1)	18 (5.0)	4 (5.7)	3 (6.5)	0 (0.0)	1 (7.1)
	25–34	74 (17.3)	63 (17.6)	11 (15.7)	10 (21.7)	1 (11.1)	0 (0.0)
	35–44	129 (30.1)	105 (29.3)	24 (34.3)	17 (37.0)	2 (22.2)	5 (35.7)
	45–54	121 (28.3)	102 (28.5)	19 (27.1)	12 (26.1)	3 (33.3)	3 (21.4)
	≥ 55	82 (19.2)	70 (19.6)	12 (17.1)	4 (8.7)	3 (33.3)	5 (35.7)
Gender, n (%)	Female	270 (63.1)	218 (60.9)	52 (74.3)	32 (69.6)	8 (88.9)	11 (78.6)
Civil status, n (%)	Single	63 (14.8)	55 (15.4)	8 (11.4)	5 (10.9)	2 (22.2)	1 (7.1)
	Married/cohabitant	244 (57.1)	205 (57.3)	39 (55.7)	28 (60.9)	4 (44.4)	6 (42.9)
	Divorced/separated	36 (8.4)	28 (7.8)	8 (11.4)	4 (8.7)	2 (22.2)	2 (14.3)
	Widower	84 (19.7)	69 (19.3)	15 (21.4)	9 (19.6)	1 (11.1)	5 (35.7)
Highest education level^b^, n (%)	No formal schooling or less than primary school	152 (35.8)	125 (35.2)	27 (38.6)	19 (41.3)	3 (33.3)	5 (35.7)
	Primary school completed	221 (52.0)	181 (51.0)	40 (57.1)	24 (52.2)	6 (66.7)	9 (64.3)
	High school or above completed	52 (12.2)	49 (13.8)	3 (4.3)	3 (6.5)	0 (0.0)	0 (0.0)
Occupation^c^, n (%)	Self-employed	108 (25.4)	94 (26.4)	14 (20.3)	9 (20.0)	2 (22.2)	2 (14.3)
	Farmer	146 (34.4)	117 (32.9)	29 (42.0)	16 (35.6)	4 (44.4)	9 (64.3)
	Other (including government and non-government employee, student, other)	82 (19.3)	74 (20.8)	8 (11.6)	6 (13.3)	0 (0.0)	2 (14.3)
	Unemployed (including volunteer, homemaker, or retired)	89 (20.9)	71 (19.9)	18 (26.1)	14 (31.1)	3 (33.3)	1 (7.1)
Socioeconomic category (Ubudehe), n (%)	1	44 (10.3)	31 (8.7)	13 (18.6)	9 (19.6)	1 (11.1)	3 (21.4)
	2	194 (45.3)	162 (45.3)	32 (45.7)	22 (47.8)	6 (66.7)	4 (28.6)
	3 or 4	186 (43.5)	161 (45.0)	25 (35.7)	15 (32.6)	2 (22.2)	7 (50.0)
	Unknown	4 (0.9)	4 (1.1)	0 (0.0)	0 (0.0)	0 (0.0)	0 (0.0)
Monthly income (RWF), median [IQR]^d^		20,000 [0, 0–50]	20,000 [0, 0–50]	10,000 [0, 0–30]	10,000 [0, 0–30]	6000 [0, 0–30]	20,000 [2750–43,750]
HIV-related data							
DSDM category, n (%)	Stable A1	318 (74.3)	277 (77.4)	41 (58.6)	28 (60.9)	5 (55.6)	7 (50.0)
	Stable A2	53 (12.4)	39 (10.9)	14 (20.0)	8 (17.4)	2 (22.2)	4 (28.6)
	Stable B	15 (3.5)	11 (3.1)	4 (5.7)	2 (4.3)	2 (22.2)	0 (0.0)
	Unstable	42 (9.8)	31 (8.7)	11 (15.7)	8 (17.4)	0 (0.0)	3 (21.4)
Years since HIV diagnosis, median [IQR]^e^		13.4 [9.5–17.0]	13.2 [9.4–17.0]	13.8 [10.5–17.4]	13.0 [9.5–17.1]	17.4 [13.8–17.8]	15.0 [12.3–16.4]
Years since ART initiation, median [IQR]^f^		11.4 [7.8–14.8]	11.3 [7.8–14.9]	11.5 [8.0–14.4]	11.0 [7.7–12.9]	14.6 [13.7–16.1]	11.4 [8.0–15.1]
Type of ART, n (%)	TDF/3TC/DTG^g^	203 (47.4)	171 (47.8)	32 (45.7)	21 (45.7)	3 (33.3)	7 (50.0)
	TDF/3TC/EFV^h^	113 (26.4)	95 (26.5)	18 (25.7)	12 (26.1)	2 (22.2)	4 (28.6)
	Other	112 (26.2)	92 (25.7)	20 (28.6)	13 (28.3)	4 (44.4)	3 (21.4)
Virally suppressed at last visit at HIV clinic^i^, n (%)	Yes	412 (96.9)	345 (97.2)	67 (95.7)	44 (95.7)	9 (100.0)	13 (92.9)
Time since last visit at HIV clinic (years), median [IQR]^j^		0.8 [0.5–1.1]	0.8 [0.5–1.1]	0.7 [0.3–1.0]	0.6 [0.3–1.0]	0.8 [0.4–1.1]	0.6 [0.3–0.9]
CD4 count from enrolment, median [IQR]^k^		369.0 [222.0–554.0]	364 [222.0–530.8]	393.0 [229.0–633.0]	408.0 [320.0–622.5]	243.5 [91.3–445.3]	439.0 [163.0–656.0]
Adherent to ART since last visit at HIV clinic, n (%)	Yes	424 (99.1)	354 (98.9)	70 (100.0)	46 (100.0)	9 (100.0)	14 (100.0)
Adherent to ART at previous visits at HIV clinic, n (%)	Yes	425 (99.3)	355 (99.2)	70 (100.0)	46 (100.0)	9 (100.0)	14 (100.0)
Psychosocial data							
Has person(s) to trust, n (%)	Yes	402 (93.9)	334 (93.3)	68 (97.1)	45 (97.8)	8 (88.9)	14 (100.0)
Family history of mental illness, n (%)	Yes	45 (10.5)	29 (8.1)	16 (22.9)	13 (28.3)	0 (0.0)	3 (21.4)
Self-reported mental health symptoms or disorders before HIV diagnosis^l^, n (%)	Yes	8 (1.9)	4 (1.1)	4 (5.7)	2 (4.3)	1 (11.1)	1 (7.1)
Self-reported mental health symptoms or disorders after HIV diagnosis^m^, n (%)	Yes	20 (4.7)	8 (2.2)	12 (17.4)	7 (15.6)	1 (11.1)	4 (28.6)
Currently or previously in treatment for mental health problems^n^, n (%)	Yes	11 (2.6)	6 (1.7)	5 (7.2)	5 (11.1)	0 (0.0)	0 (0.0)
Negative feelings related to HIV status^o^, n (%)	Yes	45 (10.6)	25 (7.0)	20 (29.0)	11 (24.4)	3 (33.3)	6 (42.9)
HIV status has impacted the ability to engage in social relationships negatively, n (%)	Yes	82 (19.2)	59 (16.5)	23 (32.9)	18 (39.1)	2 (22.2)	3 (21.4)
HIV status has impacted sexual life negatively, n (%)	Yes	50 (11.7)	33 (9.2)	17 (24.3)	14 (30.4)	1 (11.1)	2 (14.3)
Experiences of HIV stigma/discrimination, n (%)	Yes	86 (20.1)	58 (16.2)	28 (40.0)	18 (39.1)	5 (55.6)	5 (35.7)
If yes to above: HIV stigma and discrimination have affected mental well-being negatively, n (%)	Yes	39 (45.3)	20 (34.5)	19 (67.9)	12 (66.7)	3 (60.0)	4 (80.0)

Data on “ever changed ART treatment”, “previous type of ART”, “reason for ART change”, and “virally suppressed 6 months after ART initiation” are presented in Table 1a supplementary

^a^One participant was left out of the subgroup analysis as this participant had GAD without PTSD and/or MDE

^b^3 (0.70%) missing

^c^3 (0.70%) missing

^d^1(0.23%) missing

^e^2 (0.47%) missing

^f^1 (0.23%) missing

^g^Tenofovir/lamivudine/dolutegravir

^h^Tenofovir/lamivudine/efavirenz

^i^3 (0.70%) missing

^j^4 (0.93%) missing

^k^11 (2.57%) missing

^l^Self-reported symptoms/disorders include e.g. depression, anxiety, PTSD, sleeping problems, self-harm, regular and harmful use of isolation

^m^3 (0.70%) missing

^n^3 (0.70%) missing

^o^3 (0.70%) missing

### ART Adherence and Viral Suppression

The majority of the participants (n = 318, 74.3%) were in DSDM category stable A1. Practically all of the participants had been ART-adherent since their last visit to the HIV clinic (n = 424, 99.1%) and were virally suppressed (n = 412, 96.9%); this was also the case among PLWH with a mental health diagnosis. Of the 42 PLWH in the unstable DSDM category, 38 (90.5%) were virally suppressed.

### Psychosocial Evaluation

Ten percent (n = 45) of all participants reported a family history of mental illness, while this prevalence was more than doubled among participants diagnosed with any mental health disorder (n = 16/70, 22.9%) or MDE (n = 13/46, 28.3%). Among all participants, 10.6% (n = 45) reported negative feelings related to their HIV status, 19.2% (n = 82) found that their HIV status had had a negative impact on social relationships, and 11.7% (n = 50) mentioned that their HIV diagnosis had had a negative impact on their sexual life. HIV-related stigma and discrimination was experienced by 20.1% (n = 86) of the PLWH of which almost half reported that the stigmatization had affected their mental well-being negatively. For all these variables, the prevalence was higher among PLWH diagnosed with a mental health disorder, particularly MDE.

### Prevalence of Mental Health Disorders

Of the 428 PLWH included, 16.4% (n = 70) were diagnosed with at least one of the mental health disorders MDE, PTSD, and GAD. MDE was the most prevalent diagnosis (n = 60, 14.0%) followed by PTSD (n = 23, 5.4%) and GAD (n = 10, 2.3%). Around 3% (n = 14) were diagnosed with having both MDE and PTSD concomitantly, while 10.7% (n = 46) had MDE only and 2.1% (n = 9) had PTSD only. All except one of the ten PLWH with GAD had GAD as a comorbidity to their MDE or PTSD diagnosis. The prevalence of MDE and PTSD among PLWH in this study was compared to the general Rwandan population in the RMHS 2018 [45], where the population-wide prevalence of MDE and PTSD was found to be 12.0% and 3.6%, respectively. A trend towards an elevated risk for MDE was found for the PLWH in this study, albeit not being statistically significant (RR = 1.17, 95% CI 0.92–1.48, χ^2^ = 1.43, p = 0.23). For PTSD, the risk among PLWH compared to the background population was 49% higher (RR = 1.49, 95% CI 1.0–2.24, χ^2^ = 3.27, p = 0.071), but also not statistically significant.

### Awareness of Mental Health Status

Among participants diagnosed with a mental health disorder in this study, only 5.7% (n = 4/70) and 17.4% (n = 12/69) self-reported that they had experienced symptoms or received a diagnosis of mental health disorders before or after their HIV diagnosis respectively. These numbers were even lower among participants diagnosed with MDE only, where 4.3% (n = 2/46) and 15.6% (n = 7/45) reported symptoms or a diagnosis of mental health disorders before or after their HIV diagnosis. Likewise, only around 7% (n = 5/70) of participants diagnosed with MDE, PTSD, or GAD reported that they were previously or currently receiving treatment for their mental health condition.

### Factors Associated with Mental Health Disorders

Table 2 shows risk factors for having any diagnosis (MDE, PTSD, or GAD) and MDE only, while risk factors associated with the subcategories “MDE and PTSD” and “PTSD only” are presented in Supplementary Table 2a. The multivariable model included the variables age group, gender, socioeconomic class, DSDM category, family history of mental illness, self-reported mental health symptoms or diagnosis, if HIV status had a negative impact on social relations or sexual life, and HIV-related stigma and discrimination.

**Table 2:** Risk factors for having any mental health disorder (MDE, GAD, or PTSD) and MDE only compared to no diagnosis

		Any diagnosis (n = 70)				MDE only (n = 46)			
		RR (95% CI)	p-value	aRR (95% CI)	p-value	RR (95% CI)	p-value	aRR (95% CI)	p-value
Sociodemographic data									
Age group	≤ 24	Ref.	Ref.	Ref.	Ref.	Ref.	Ref.	Ref.	Ref.
	25–34	0.82 (0.29–2.31)	0.74	1.40 (0.53–3.72)	0.50	0.96 (0.29–3.17)	1.00	1.28 (0.48–3.45)	0.63
	35–44	1.02 (0.39–2.66)	1.00	1.66 (0.66–4.13)	0.28	0.98 (0.31–3.04)	1.00	1.22 (0.48–3.08)	0.67
	45–54	0.86 (0.32–2.30)	0.76	1.38 (0.54–3.52)	0.50	0.74 (0.23–2.39)	0.70	0.94 (0.36–2.46)	0.90
	≥ 55	0.80 (0.29–2.25)	0.74	1.21 (0.45–3.24)	0.71	0.38 (0.09–1.56)	0.18	0.54 (0.15–1.97)	0.35
Gender	Female (vs male)	1.69 (1.03–2.78)	0.042	1.35 (0.81–2.22)	0.25	1.41 (0.78–2.55)	0.33	0.94 (0.51–1.73)	0.85
Civil status	Single	Ref.	Ref.			Ref.	Ref.		
	Married or cohabitant	1.26 (0.62–2.56)	0.69			1.44 (0.58–3.58)	0.50		
	Divorced or separated	1.75 (0.72–4.26)	0.26			1.50 (0.43–5.20)	0.71		
	Widower	1.41 (0.53–3.11)	0.49			1.38 (0.49–3.92)	0.58		
Educational level	No formal schooling or less than primary school	Ref.	Ref.			Ref.	Ref.		
	Primary school completed	1.02 (0.65–1.59)	1.00			0.89 (0.51–1.56)	0.74		
	High school or above completed	0.32 (0.10–1.03)	0.041			0.44 (0.13–1.42)	0.20		
Occupation	Other (including government and non-government employee, student, other)	Ref.	Ref.			Ref.	Ref.		
	Self-employed	1.33 (0.59–3.02)	0.65			1.17 (0.43–3.14)	0.79		
	Unemployed (including volunteer, homemaker, or retired)	2.07 (0.95–4.51)	0.087			2.20 (0.89–5.44)	0.10		
	Farmer	2.04 (0.98–4.24)	0.061			1.60 (0.65–3.93)	0.36		
Socioeconomic category (Ubudehe)	1	Ref.	Ref.	Ref.	Ref.	Ref.	Ref.	Ref.	Ref.
	2	0.56 (0.32–0.97)	0.056	0.72 (0.40–1.30)	0.27	0.53 (0.26–1.07)	0.13	0.66 (0.31–1.41)	0.29
	3 or 4	0.45 (0.25–0.82)	0.014	0.81 (0.42–1.56)	0.52	0.37 (0.18–0.80)	0.022	0.64 (0.28–1.48)	0.30
HIV-related data									
DSDM category	Stable A1	Ref.	Ref.	Ref.	Ref.	Ref.	Ref.	Ref.	Ref.
	Stable A2	2.05 (1.20–3.49)	0.019	1.55 (0.91–2.64)	0.11	1.85 (0.90–3.82)	0.12	1.27 (0.62–2.60)	0.51
	Stable B	2.07 (0.85–5.02)	0.13	1.71 (0.62–4.73)	0.30	1.68 (0.45–6.29)	0.35	1.17 (0.36–3.80)	0.79
	Unstable	2.03 (1.13–3.64)	0.033	2.00 (1.08–3.71)	0.027	2.23 (1.10–4.55)	0.046	2.14 (0.96–4.77)	0.063
Type of ART	TDF/3TC/DTG	Ref.	Ref.			Ref.	Ref.		
	TDF/3TC/EFV	1.01 (0.59–1.72)	1.00			1.03 (0.53–2.00)	1.00		
	Other	1.13 (0.68–1.88)	0.64			1.13 (0.59–2.17)	0.71		
Virally suppressed at last visit	Yes (vs no)	0.70 (0.26–1.95)	0.46			0.68 (0.19–2.48)	0.64		
Virally suppressed at visit 6 months after ART initiation	Yes (vs no)	0.73 (0.40–1.34)	0.30			0.70 (0.33–1.47)	0.33		
Psychosocial data									
Has person(s) to trust	No (vs yes)	0.45 (0.12–1.75)	0.28			0.34 (0.05–2.34)	0.34		
Family history of mental illness	Yes (vs no)	2.52 (1.58–4.01)	0.00089	1.81 (1.08–3.03)	0.025	3.40 (1.95–5.92)	0.00023	2.54 (1.35–4.75)	0.0037
Self-reported symptoms/diagnosis of mental health disorders before HIV diagnosis	Yes (vs no)	3.24 (1.56–6.72)	0.026	2.60 (0.91–7.44)	0.076	3.12 (0.97–10.02)	0.13	1.58 (0.37–6.64)	0.53
Self-reported symptoms/diagnosis of mental health disorders after HIV diagnosis	Yes (vs no)	4.26 (2.77–6.56)	< 0.0001			4.74 (2.55–8.81)	0.00046		
Currently or previously in treatment for mental health problems	Yes (vs no)	2.94 (1.48–5.84)	0.020			4.43 (2.18–9.02)	0.0039		
Negative feelings related to HIV status	Yes (vs no)	3.45 (2.27–5.24)	< 0.0001			3.28 (1.82–5.90)	0.00077		
HIV status has impacted the ability to engage in social relationships negatively	Yes (vs no)	2.09 (1.35–3.24)	0.0024	1.01 (0.61–1.69)	0.96	2.81 (1.63–4.82)	0.00049	1.34 (0.73–2.46)	0.35
HIV status has impacted sexual life negatively	Yes (vs no)	2.44 (1.54–3.87)	0.00088	1.78 (1.02–3.12)	0.042	3.38 (1.95–5.88)	0.00017	1.98 (0.98–4.04)	0.059
Experiences of HIV stigma/discrimination	Yes (vs no)	2.68 (1.77–4.08)	< 0.0001	2.14 (1.30–3.53)	0.0030	2.84 (1.65–4.88)	0.00044	2.13 (1.13–4.02)	0.019
If yes to above: HIV stigma and discrimination have affected mental well-being negatively	Yes (vs no)	2.54 (1.30–4.97)	0.0053			2.75 (1.15–6.55)	0.027		

Multivariable model is adjusted for age group, gender, socioeconomic class, DSDM category, family history of mental illness, self-reported mental health symptoms or diagnosis, if HIV status had negative impact on social relations or sexual life, and HIV-related stigma and discrimination

Data for PTSD and MDE and PTSD only are shown in Table 2a supplementary

Among the PLWH diagnosed with any mental health disorder, female participants were at increased risk of mental health disorders (RR = 1.69, 95% CI 1.03–2.78, p = 0.042), but the association was not significant in the adjusted model (aRR = 1.35, 95% CI 0.81–2.22, p = 0.25) (Table 2). The risk of mental health disorders was inversely associated with educational level, with the risk being lower among PLWH with higher education levels (high school or above completed, i.e. participants who have completed secondary school, college/university, or a post-graduate degree) (RR = 0.32, 95% CI 0.10–1.03, p = 0.041). Similarly, PLWH in higher socioeconomic classes showed a reduced risk (RR = 0.45, 95% CI 0.25–0.82, p = 0.014) of mental health disorder. However, the result was only statistically significant for socioeconomic class 3 and 4 and not in the adjusted model.

On the contrary, family history of mental illness was increasing the risk of any disorder by 80% (aRR = 1.81, 95% CI 1.08–3.03, p = 0.025) and more for those with MDE alone (aRR = 2.54, 95% CI 1.35–4.75, p = 0.0037). The risk of having a mental health disorder was also significantly higher among PLWH in the DSDM unstable category (i.e., clients with unsuppressed viral load, on 3rd line ART, newly initiated ART, breastfeeding, acute malnutrition, or unstable mental disorders) (aRR = 2.00, 95% CI 1.08–3.71, p = 0.027). Participants who reported negative feelings related to their HIV status were at increased risk of suffering from any of the assessed mental health disorders (RR = 3.45, 95% CI 2.27–5.24, p < 0.0001), but also of having either MDE only, MDE and PTSD, or PTSD only. Likewise, participants who reported that their HIV diagnosis had had a negative impact on their sexual life were at increased risk of having any mental health disorder (aRR = 1.78, 95% CI 1.02–3.12, p = 0.042). This was also the case for PLWH reporting experiences of HIV-related stigma and discrimination who had a significantly higher risk of mental illnesses in general (aRR = 2.14, 95% CI 1.30–3.53, p = 0.0030) and for being diagnosed with MDE only (aRR = 2.13, 95% CI 1.13–4.02, p = 0.019) or PTSD only (aRR = 10.51, 95% CI 2.32–47.60, p = 0.0023). Lastly, participants responding that their HIV diagnosis had had a negative impact on social relations were at an increased risk of any mental health disorder (RR = 2.09, 95% CI 1.35–3.24, p = 0.0024), but this association was not significant in the adjusted model (aRR = 1.01, 95% CI 0.61–1.69, p = 0.96).

## Discussion

To our knowledge, this study is the first to assess the prevalence of MDE, PTSD, and GAD and associated risk factors among a sample of PLWH from Rwanda representing the general HIV population of Rwanda with regards to age, gender, and geographical location (rural or urban residence). The prevalence of MDE, PTSD, and GAD among the Rwandan PLWH were estimated to be 14.0%, 5.4%, and 2.3%, respectively. Around three percent were diagnosed with PTSD and MDE concomitantly. Surprisingly, it was found that the participants were almost completely adherent to ART and fully virally suppressed regardless of their mental health diagnosis, and also among PLWH in the unstable DSDM category. Among others, HIV-related stigma and discrimination were associated with an increased risk of mental health disorders. Only 5% and 17% of the PLWH diagnosed with a mental health disorder in this study self-reported mental health symptoms or diagnosis prior to or after receiving their HIV diagnosis respectively, and only 7% of the participants with a diagnosis were receiving treatment. These results indicate that among PLWH in Rwanda, mental health disorders are underdiagnosed and undertreated.

### The Burden of Mental Health Disorders Among PLWH

It has previously been shown that PLWH are more likely to suffer from mental health disorders than the general population [10, 12–14]. In this study, the burden of MDE and PTSD among PLWH was higher than in the Rwandan general population, but the difference was statistically non-significant.

A potential explanation for not detecting a significant difference in the prevalence of mental health disorders among PLWH and the Rwandan population might be an underestimation of the actual prevalence of MDE and PTSD among PLWH in Rwanda due to a short time span of recruitment, the clinic-based recruitment, and the exclusion of PLWH with certain chronic conditions and pregnant women, which potentially could be at higher risk for mental health disorders in general. Yet, the results from this study are consistent with a Rwandan study in which 11% of the PLWH were diagnosed with depression [46] and previous studies from other LMICs in sub-Saharan Africa showing depression prevalence rates of 15–20% among PLWH [9, 24]. However, another Rwandan study found a depression rate of 47% among PLWH [47], but only including rural settings in contrast to the both rural and urban areas covered in this study.

An additional explanation for not detecting a larger difference in the prevalence of mental health disorders among PLWH and the general population could be the Genocide against the Tutsi, resulting in a burden of mental health disorders in the general population of Rwanda, which has been estimated to 20.5% [45]. This might explain that the differences between PLWH and the general population may not be as distinct as in other countries. Nevertheless, studies conducted in other sub-Saharan African countries have identified prevalence rates of mental health disorders within the general population equal to or higher than the prevalence found in Rwanda, albeit using other instruments than the MINI. A systematic review and meta-analysis in Uganda found a pooled prevalence of any mental disorder among the population of 24.2% [55], whereas South African citizens were found with a prevalence of 16.5% [56]. Consequently, it appears that the prevalence of mental health disorders among the Rwandan population is not significantly elevated compared to other sub-Saharan African countries, but the methodological variations of the literature challenge comparison.

### Effect on ART Adherence and Viral Suppression

The high ART adherence among PLWH with mental illness in this study contrasts with results from other studies in LMICs. In a recent South African study, depression and anxiety were associated with a risk of non-adherence [17], and a meta-analysis of studies from Sub-Saharan Africa showed 55% lower odds of achieving good ART adherence among PLWH with depression or depressive symptoms compared to participants without [23].

In our study, information on ART adherence was collected retrospectively as a part of the interview with participants self-reporting their adherence at the last clinical visit, which could lead to recall bias. However, the HIV nurses had access to patient files, where the information could be crosschecked which together with the overall suppressed viral load increases the validity of the adherence data.

The high ART adherence found in the study may be a result of the WHO-recommended DSDM [52, 53], which has been successfully implemented in Rwanda. Rwanda’s National HIV programme has previously been documented to be efficient in ensuring high ART adherence [57]. Still, the association between mental health disorders and ART adherence may exist in other national settings with less HIV quality care and support.

### Risk Factors for Mental Disorders

Consistent with previous reports [45, 58], educational level and experiences of stigma and discrimination were associated with mental health disorders. Likewise, being in the unstable DSDM category was also a risk factor. These factors may be used to identify vulnerable PLWH in particular need of screening for mental health disorders. PLWH reporting negative feelings related to their HIV status or stating that HIV diagnosis had negative impact on their sexual life and relationships also had a higher risk of being diagnosed in this study. However, the nature of the study’s cross-sectional design precludes the determination of causal associations between risk factors and mental health disorders. For several of the above-mentioned factors, reverse causality may exist. Nevertheless, the fact that these associations were found still underlines the need for bringing mental health among PLWH into focus.

Around 20% of the participants in this study reported experiences of stigma and discrimination, of which almost half reported that the stigmatization had negatively affected their mental health. This underlines that stigmatization of PLWH is still a significant challenge that requires attention to increase the quality of life of PLWH, also in low-resource settings. However, the prevalence of reported stigma is remarkably low as compared to what has previously been shown in South Africa, where stigmatizing experiences among PLWH ranged from 43.5 to 88% [59]. The inconsistency may be due to methodological differences; in this study, a non-validated question was used to assess any kind of HIV-related stigma or discrimination rather than a validated stigma score, and this could have underestimated the actual prevalence. This, along with the tabooed nature of the question, could also explain why only 11.7% of the participants reported that HIV had negatively affected their sexual life in this study. However, in a recent report on stigma among PLWH in Rwanda only around 30% of the participants reported any form of stigma [60]. Thus, the experience of stigma among PLWH may in general be lower in Rwanda than e.g. South Africa; maybe because most Rwandan PLWH have a well-controlled HIV infection [60], and, thus, may have a lower risk of being subject to discrimination.

### Integrating Mental Health with HIV Care

The results indicate that among PLWH in Rwanda, mental health disorders are underdiagnosed and undertreated. Previous studies have also highlighted the undertreatment of mental health disorders in LMIC [26] due to the lack of specialist mental health care and multidisciplinary workforce providing psychosocial support [27]. It was recently estimated that only 62% of the Rwandan general population were aware of mental health services and only 5% utilized these, where the main reason for not seeking help was a lack of perceived need for treatment of mental health symptoms [45]. Additionally, a recently published study of people with bipolar disorder in Rwanda found that 44% were not aware that they suffered from bipolar disorder [61]. Thus, concurrently with the limited accessibility, the demand for mental health services may also be low in low-resource settings due to low awareness and literacy, stigma, or simply due to a misinterpretation of mental health symptoms as reactions to social or economic stressors not requiring treatment rather than actual health problems [62]. Integrating mental health care into primary care in LMICs has previously been shown to be efficient [63], and considering the well-established HIV programme in many LMICs [64], implementing mental health care with the existing national HIV services may not only resolve the problem of underdiagnosis of mental health disorders among PLWH, but could also serve as a way of raising awareness to detect mental health symptoms at an early stage as a part of a preventive strategy [48, 58, 65]. Our study is an example of this approach by training HIV nurses in screening PLWH for mental health disorders.

### Strengths and Limitations of the Study

The cross-sectional study design hinders the determination of causality between risk factors and the mental health disorders. Determining significant associations is also limited by the sample size, and, thus, the associations explored calls for further investigation in larger studies. In this study, no genocide-related data was collected, but given the age of the participants, it is likely that a part of the mental burden could be explained by genocide trauma rather than HIV. Mental trauma is known to increase the risk of a wide range of mental health problems [66]. A recent population-wide survey of the mental health status in Rwanda found MDE to be three times higher among genocide survivors compared to the general population [45]. Likewise, future studies would also benefit from including other vulnerable groups such as PLWH with substance and alcohol abuse which are associated with mental health disorders [10]. Also, looking at PLWH with same-sex partners or sex workers would add valuable information on the prevalence of mental health disorders among these populations, which presumably are at higher risk of experiencing stigma and discrimination.

The inclusion of PLWH to represent the general HIV population of Rwanda with regard to gender and age together with the large study area representing both rural and urban Rwanda adds to the representativeness and external validity of the study. Among the selected PLWH in our study, reasons for non-participation were limited to either death, not being able to contact the patient, or the patient having moved to another province. Thus, the risk of selection bias arising from this study is expected to be low, but the study still has certain limitations related to the inclusion process, which makes the population less representative as compared to the RMHS. In the original cohort, PLWH who were not committed to long-term follow-up, who were pregnant or < 3 months post-partum, with self-reported cardiovascular disease, diabetes, cancer or clinically confirmed hypertension were excluded, and the PLWH participating may therefore be more well-functioning and with fewer comorbidities than non-participants. As loss-to-follow-up in the original cohort from which the participants were enrolled is unknown, it cannot be ruled out that non-participating PLWH have certain common characteristics or risk factors which could result in selection bias. While PLWH with certain chronic conditions have been excluded from this study, the above-mentioned exclusion criteria did not apply to the RMHS, where eligible participants were Rwandan citizens aged 14–65 years living in Rwanda at the time of the survey, who had been living in their respective areas for at least 6 months, and with sufficient communication abilities to complete the interview. Likely, the prevalence of mental health disorders is elevated among people with chronic illnesses, which have been excluded from our study, thereby leading to a potential underestimation of the prevalence of mental health disorders in our study. This might explain why only marginal differences in mental health disorder prevalence were detected compared to the RMHS.

Bias could also have been introduced by only recruiting PLWH who already consented to participate in the original study, as these PLWH may represent only the most socioeconomically advantaged part of this group with fewer comorbidities. The short enrolment period of only a few months may have caused an underestimation of the actual prevalence of MDE, PTSD, and GAD since PLWH with a more significant mental health burden might not have been able to access the clinic during that timeframe. Similarly, the choice of clinic-based recruitment rather than an outreach approach may have led to the exclusion of the PLWH mostly affected by mental health disorders as they might not have been able to attend the clinic. Furthermore, the sample size calculation was only based on the prevalence of MDE among the background population of Rwanda as estimated in the RMHS [45], since MDE was the most prevalent mental health disorder among the Rwandan population. Due to this, it cannot be ruled out that the actual prevalence of PTSD or GAD among PLWH of Rwanda has been underestimated, and whether the difference in the prevalence of PTSD among PLWH as compared to the background population could have been statistically significant if a larger sample size had been used.

To further increase the generalizability, future studies should also include children and adolescents with HIV among whom mental health disorders have previously been shown to be a widespread problem [8]. Since no control group was included, the results could only be compared to previous results from the general population in the RMHS [45], which could have introduced bias due to the temporal difference in the data collection. Using Kinyarwanda-speaking trained HIV nurses to conduct the previously validated MINI [49, 50] contributes to the validity and reliability of the diagnoses. Inter-interviewer variability can, however, not be precluded in the assessment of the participants’ sociodemographic and psychosocial information, where a non-validated questionnaire was used, but the common training of the interviewers prior to the interviews ensures accuracy of the results.

## Conclusion

The results confirm previously reported high rates of MDE, PTSD, and GAD among PLWH. Experiences of stigma and discrimination were associated with increased risk of mental health disorders, while a high educational level was a protective factor. This underlines the importance of identifying vulnerable groups to be screened for mental disorders. However, no association with adherence to ART was found as seen in other studies, which may be explained by the well-functioning HIV management program in Rwanda. Underdiagnosis and lacking awareness of mental health disorders was a widespread problem among the participants highlighting the need for increased mental health accessibility, awareness, and literacy among PLWH and in the Rwandan general population. By applying a low-cost approach to diagnose mental health disorders through community health workers and using the MINI, this study design could serve as an example of integrating mental health diagnostics with existing HIV or other chronic disease care. This integration approach has broad implications not only in LMICs but globally and should be further investigated in larger studies and in other settings to inspire policymakers to tackle the burden of mental disorders among PLWH.

### Supplementary Information

Below is the link to the electronic supplementary material.

Supplementary file1 (DOCX 36 KB)

## Data Availability

Data is available on request.

## References

[1] A. UNAIDS. Global factsheets 2021 HIV and AIDS estimates. https://aidsinfo.unaids.org. Accessed 27 Apr 2023.

[2] Hogg RS, et al. Improved survival among HIV-infected individuals following initiation of antiretroviral therapy. JAMA. 1998;279(6):450–4. 10.1001/jama.279.6.450. (**in English**). 9466638 10.1001/jama.279.6.450

[3] Mocroft A, et al. Decline in the AIDS and death rates in the EuroSIDA study: an observational study. Lancet. 2003;362(9377):22–9. 10.1016/s0140-6736(03)13802-0. (**in English**). 12853195 10.1016/s0140-6736(03)13802-0

[4] Kaluvu L, et al. Multimorbidity of communicable and non-communicable diseases in low- and middle-income countries: a systematic review. J Multimorb Comorb. 2022. 10.1177/26335565221112593. (**in English**). 36081708 10.1177/26335565221112593 PMC9445468

[5] Jespersen NA, Axelsen F, Dollerup J, Nørgaard M, Larsen CS. The burden of non-communicable diseases and mortality in people living with HIV (PLHIV) in the pre-, early- and late-HAART era. HIV Med. 2021;22(6):478–90. 10.1111/hiv.13077. (**in English**). 33645000 10.1111/hiv.13077 PMC8247855

[6] Schouten J, et al. Cross-sectional comparison of the prevalence of age-associated comorbidities and their risk factors between HIV-infected and uninfected individuals: the AGEhIV cohort study. Clin Infect Dis. 2014;59(12):1787–97. 10.1093/cid/ciu701. (**in English**). 25182245 10.1093/cid/ciu701

[7] Hirschhorn LR, Kaaya SF, Garrity PS, Chopyak E, Fawzi MC. Cancer and the “other” noncommunicable chronic diseases in older people living with HIV/AIDS in resource-limited settings: a challenge to success. AIDS. 2012;26(Suppl 1):S65-75. 10.1097/QAD.0b013e328355ab72. (**in English**). 22781178 10.1097/QAD.0b013e328355ab72

[8] Benton TD, KeeNg WY, Leung D, Canetti A, Karnik N. Depression among youth living with HIV/AIDS. Child Adolesc Psychiatr Clin N Am. 2019;28(3):447–59. 10.1016/j.chc.2019.02.014. (**in English**). 31076119 10.1016/j.chc.2019.02.014

[9] Parcesepe AM, et al. Gender, mental health, and entry into care with advanced HIV among people living with HIV in Cameroon under a national “treat all” policy. AIDS Behav. 2021;25(12):4018–28. 10.1007/s10461-021-03328-3. (**in English**). 34091803 10.1007/s10461-021-03328-3 PMC8938985

[10] Hoare J, Sevenoaks T, Mtukushe B, Williams T, Heany S, Phillips N. Global systematic review of common mental health disorders in adults living with HIV. Curr HIV/AIDS Rep. 2021. 10.1007/s11904-021-00583-w. 34792706 10.1007/s11904-021-00583-w PMC8600343

[11] Ayano G, Demelash S, Abraha M, Tsegay L. The prevalence of depression among adolescent with HIV/AIDS: a systematic review and meta-analysis. AIDS Res Ther. 2021;18(1):23. 10.1186/s12981-021-00351-1. (**in English**). 33906698 10.1186/s12981-021-00351-1 PMC8077927

[12] Hémar V, et al. A comprehensive analysis of excess depressive disorder in women and men living with HIV in France compared to the general population. Sci Rep. 2022;12(1):6364. 10.1038/s41598-022-10263-3. (**in English**). 35430622 10.1038/s41598-022-10263-3 PMC9013369

[13] Morales DR, Moreno-Martos D, Matin N, McGettigan P. Health conditions in adults with HIV compared with the general population: a population-based cross-sectional analysis. EClinicalMedicine. 2022;47:101392. 10.1016/j.eclinm.2022.101392. (**in English**). 35497059 10.1016/j.eclinm.2022.101392 PMC9046106

[14] Do AN, et al. Excess burden of depression among HIV-infected persons receiving medical care in the united states: data from the medical monitoring project and the behavioral risk factor surveillance system. PLoS ONE. 2014;9(3): e92842. 10.1371/journal.pone.0092842. (**in English**). 24663122 10.1371/journal.pone.0092842 PMC3963963

[15] Tao J, Vermund SH, Qian HZ. Association between depression and antiretroviral therapy use among people living with HIV: a meta-analysis. AIDS Behav. 2018;22(5):1542–50. 10.1007/s10461-017-1776-8. (**in English**). 28439754 10.1007/s10461-017-1776-8 PMC7942230

[16] Mayston R, Kinyanda E, Chishinga N, Prince M, Patel V. Mental disorder and the outcome of HIV/AIDS in low-income and middle-income countries: a systematic review. AIDS. 2012;26(Suppl 2):S117–35. 10.1097/QAD.0b013e32835bde0f. (**in English**). 23303434 10.1097/QAD.0b013e32835bde0f

[17] Haas AD, et al. Mental health, ART adherence, and viral suppression among adolescents and adults living with HIV in South Africa: a cohort study. AIDS Behav. 2023. 10.1007/s10461-022-03916-x. (**in English**). 36592251 10.1007/s10461-022-03916-x PMC10149479

[18] Evans DL, et al. Association of depression with viral load, CD8 T lymphocytes, and natural killer cells in women with HIV infection. Am J Psychiatry. 2002;159(10):1752–9. 10.1176/appi.ajp.159.10.1752. (**in English**). 12359683 10.1176/appi.ajp.159.10.1752

[19] Bengtson AM, et al. Depressive symptoms and engagement in human immunodeficiency virus care following antiretroviral therapy initiation. Clin Infect Dis. 2019;68(3):475–81. 10.1093/cid/ciy496. (**in English**). 29901695 10.1093/cid/ciy496 PMC6336906

[20] Bouhnik AD, et al. Depression and clinical progression in HIV-infected drug users treated with highly active antiretroviral therapy. Antivir Ther. 2005;10(1):53–61 (**in English**). 15751763 10.1177/135965350501000103

[21] Kaharuza FM, et al. Depression and CD4 cell count among persons with HIV infection in Uganda. AIDS Behav. 2006;10(4 Suppl):S105–11. 10.1007/s10461-006-9142-2. (**in English**). 16802195 10.1007/s10461-006-9142-2

[22] Too EK, et al. Prevalence and factors associated with common mental disorders in young people living with HIV in sub-Saharan Africa: a systematic review. J Int AIDS Soc. 2021;24:e25705. 10.1002/jia2.25705. (**in English**). 34164931 10.1002/jia2.25705 PMC8222842

[23] Nakimuli-Mpungu E, et al. Depression, alcohol use and adherence to antiretroviral therapy in sub-Saharan Africa: a systematic review. AIDS Behav. 2012;16(8):2101–18. 10.1007/s10461-011-0087-8. (**in English**). 22116638 10.1007/s10461-011-0087-8

[24] Marwick KF, Kaaya SF. Prevalence of depression and anxiety disorders in HIV-positive outpatients in rural Tanzania. AIDS Care. 2010;22(4):415–9. 10.1080/09540120903253981. (**in English**). 20131127 10.1080/09540120903253981

[25] HIV.gov. Global statistics the global HIV/AIDS epidemic. https://www.hiv.gov/hiv-basics/overview/data-and-trends/global-statistics/. Accessed 27 Apr 2023

[26] Thornicroft G, et al. Undertreatment of people with major depressive disorder in 21 countries. Br J Psychiatry. 2017;210(2):119–24. 10.1192/bjp.bp.116.188078. (**in English**). 27908899 10.1192/bjp.bp.116.188078 PMC5288082

[27] Hanlon C, et al. Challenges and opportunities for implementing integrated mental health care: a district level situation analysis from five low- and middle-income countries. PLoS ONE. 2014;9(2): e88437. 10.1371/journal.pone.0088437. (**in English**). 24558389 10.1371/journal.pone.0088437 PMC3928234

[28] Bruckner TA, et al. The mental health workforce gap in low- and middle-income countries: a needs-based approach. Bull World Health Organ. 2011;89(3):184–94. 10.2471/blt.10.082784. (**in English**). 21379414 10.2471/blt.10.082784 PMC3044251

[29] UNAIDS. 90–90–90: an ambitious treatment target to help end the aids epidemic. https://www.unaids.org/en/resources/909090. Accessed 1 Feb 2022.

[30] UNAIDS. 2025 AIDS targets, 25/1/2021 (2021). https://www.unaids.org/sites/default/files/2025-AIDS-Targets_en.pdf. Accessed 25 May 2022.

[31] Beer L, Tie Y, Padilla M, Shouse RL. Generalized anxiety disorder symptoms among persons with diagnosed HIV in the United States. AIDS. 2019;33(11):1781–7. 10.1097/qad.0000000000002286. (**in English**). 31211718 10.1097/qad.0000000000002286 PMC6663599

[32] Kalomo EN. Associations between HIV-related stigma, self-esteem, social support, and depressive symptoms in Namibia. Aging Ment Health. 2018;22(12):1570–6. 10.1080/13607863.2017.1387763. (**in English**). 29019412 10.1080/13607863.2017.1387763

[33] Lu H, et al. Inflammation and risk of depression in HIV: prospective findings from the Multicenter AIDS Cohort Study. Am J Epidemiol. 2019;188(11):1994–2003. 10.1093/aje/kwz190. (**in English**). 31642472 10.1093/aje/kwz190 PMC6825834

[34] MudraRakshasa-Loots A, Whalley HC, Vera JH, Cox SR. Neuroinflammation in HIV-associated depression: evidence and future perspectives. Mol Psychiatry. 2022;27(9):3619–32. 10.1038/s41380-022-01619-2. (**in English**). 35618889 10.1038/s41380-022-01619-2 PMC9708589

[35] Del Guerra FB, Fonseca JL, Figueiredo VM, Ziff EB, Konkiewitz EC. Human immunodeficiency virus-associated depression: contributions of immuno-inflammatory, monoaminergic, neurodegenerative, and neurotrophic pathways. J Neurovirol. 2013;19(4):314–27. 10.1007/s13365-013-0177-7. (**in English**). 23868513 10.1007/s13365-013-0177-7

[36] Li CW, et al. Efavirenz is not associated with an increased risk of depressive disorders in patients living with HIV: an 11-year population-based study in Taiwan. Healthcare (Basel). 2021. 10.3390/healthcare9121625. (**in English**). 34946352 10.3390/healthcare9121625 PMC8701138

[37] Gutiérrez F, et al. Risk of clinically significant depression in HIV-infected patients: effect of antiretroviral drugs. HIV Med. 2014;15(4):213–23. 10.1111/hiv.12104. (**in English**). 24215356 10.1111/hiv.12104

[38] Gaida R, Truter I, Grobler C, Kotze T, Godman B. A review of trials investigating efavirenz-induced neuropsychiatric side effects and the implications. Expert Rev Anti Infect Ther. 2016;14(4):377–88. 10.1586/14787210.2016.1157469. (**in English**). 26900637 10.1586/14787210.2016.1157469

[39] Cook JA, et al. Prevalence, comorbidity, and correlates of psychiatric and substance use disorders and associations with hiv risk behaviors in a multisite cohort of women living with HIV. AIDS Behav. 2018;22(10):3141–54. 10.1007/s10461-018-2051-3. (**in English**). 29460130 10.1007/s10461-018-2051-3 PMC6153984

[40] Abayomi O, Adelufosi A, Adebayo P, Ighoroje M, Ajogbon D, Ogunwale A. HIV risk behavior in persons with severe mental disorders in a psychiatric hospital in Ogun, Nigeria. Ann Med Health Sci Res. 2013;3(3):380–4. 10.4103/2141-9248.117960. (**in English**). 24116318 10.4103/2141-9248.117960 PMC3793444

[41] Wainberg ML, et al. HIV risk behaviors among outpatients with severe mental illness in Rio de Janeiro, Brazil. World Psychiatry. 2008;7(3):166–72. 10.1002/j.2051-5545.2008.tb00190.x. (**in English**). 18836542 10.1002/j.2051-5545.2008.tb00190.x PMC2559926

[42] UNAIDS. Country factsheets; Rwanda 2021; HIV and AIDS estimates. https://www.unaids.org/en/regionscountries/countries/rwanda. Accessed 3 May 2023.

[43] Mukamana D, Brysiewicz P, Collins A, Rosa W. Genocide rape trauma management: an integrated framework for supporting survivors. ANS Adv Nurs Sci. 2018;41(1):41–56. 10.1097/ans.0000000000000177. (**in English**). 28614103 10.1097/ans.0000000000000177

[44] Mukamana D, Brysiewicz P. The lived experience of genocide rape survivors in Rwanda. J Nurs Scholarsh. 2008;40(4):379–84. 10.1111/j.1547-5069.2008.00253.x. (**in English**). 19094154 10.1111/j.1547-5069.2008.00253.x

[45] Kayiteshonga Y, Sezibera V, Mugabo L, Iyamuremye JD. Prevalence of mental disorders, associated co-morbidities, health care knowledge and service utilization in Rwanda-towards a blueprint for promoting mental health care services in low- and middle-income countries? BMC Public Health. 1858;22(1):2022. 10.1186/s12889-022-14165-x. (**in English**). 10.1186/s12889-022-14165-x PMC9533613 36199102

[46] Wroe EB, Hedt-Gauthier BL, Franke MF, Nsanzimana S, Turinimana JB, Drobac P. Depression and patterns of self-reported adherence to antiretroviral therapy in Rwanda. Int J STD AIDS. 2015;26(4):257–61. 10.1177/0956462414535206. (**in English**). 24828554 10.1177/0956462414535206

[47] Krumme AA, Kaigamba F, Binagwaho A, Murray MB, Rich ML, Franke MF. Depression, adherence and attrition from care in HIV-infected adults receiving antiretroviral therapy. J Epidemiol Commun Health. 2015;69(3):284–9. 10.1136/jech-2014-204494. (**in English**). 10.1136/jech-2014-204494 25385745

[48] Chuah FLH, et al. Interventions and approaches to integrating HIV and mental health services: a systematic review. Health Policy Plan. 2017. 10.1093/heapol/czw169. (**in English**). 29106512 10.1093/heapol/czw169 PMC5886062

[49] Kayiteshonga Y. Rwanda Mental Health Survey 2018. Kigali: Rwanda Biomedical Center; 2018.

[50] Pettersson A, Modin S, Wahlström R, AfWinklerfeltHammarberg S, Krakau I. The Mini-International Neuropsychiatric Interview is useful and well accepted as part of the clinical assessment for depression and anxiety in primary care: a mixed-methods study. BMC Fam Pract. 2018;19(1):19. 10.1186/s12875-017-0674-5. (**in English**). 29368585 10.1186/s12875-017-0674-5 PMC5781342

[51] Rwandapedia. Ubudehe: Poverty level categories. https://rwandapedia.rw/hgs/ubudehe/poverty-level-categories. Accessed 3 May 2023.

[52] W. World Health Organization, Updated recommendations on service delivery for the treatment and care of people living with HIV. 2021. https://www.who.int/publications/i/item/9789240023581. Accessed 15 May 2023. 33999550

[53] R. B. C. Ministry of Health, Guidelines for HIV prevention, treatment and care in Rwanda. 2022. https://rbc.gov.rw/fileadmin/user_upload/guidelines%2023/Final%20GUIDELINES%20FOR%20HIV%20PREVENTION%2C%20TREATMENT%20AND%20CARE%20IN%20RWANDA%202022%20c.pdf

[54] Zou G. A modified Poisson regression approach to prospective studies with binary data. Am J Epidemiol. 2004;159(7):702–6. 10.1093/aje/kwh090. (**in English**). 15033648 10.1093/aje/kwh090

[55] Opio JN, Munn Z, Aromataris E. Prevalence of mental disorders in uganda: a systematic review and meta-analysis. Psychiatr Q. 2022;93(1):199–226. 10.1007/s11126-021-09941-8. (**in English**). 34427855 10.1007/s11126-021-09941-8

[56] Williams DR, et al. Twelve-month mental disorders in South Africa: prevalence, service use and demographic correlates in the population-based South African Stress and Health Study. Psychol Med. 2008;38(2):211–20. 10.1017/s0033291707001420. (**in English**). 17903333 10.1017/s0033291707001420 PMC2718686

[57] Elul B, et al. High levels of adherence and viral suppression in a nationally representative sample of HIV-infected adults on antiretroviral therapy for 6, 12 and 18 months in Rwanda. PLoS ONE. 2013;8(1): e53586. 10.1371/journal.pone.0053586. (**in English**). 23326462 10.1371/journal.pone.0053586 PMC3541229

[58] Remien RH, Stirratt MJ, Nguyen N, Robbins RN, Pala AN, Mellins CA. Mental health and HIV/AIDS: the need for an integrated response. AIDS. 2019;33(9):1411–20. 10.1097/qad.0000000000002227. (**in English**). 30950883 10.1097/qad.0000000000002227 PMC6635049

[59] MacLean JR, Wetherall K. The association between HIV-stigma and depressive symptoms among people living with HIV/AIDS: a systematic review of studies conducted in South Africa. J Affect Disord. 2021;287:125–37. 10.1016/j.jad.2021.03.027. (**in English**). 33780828 10.1016/j.jad.2021.03.027

[60] R. Rwanda Biomedical Centre, Rwanda People Living with HIV Index 2.0, survey report. 2020. https://www.stigmaindex.org/wp-content/uploads/2020/11/Rwanda-SI-Report-2020.pdf. Accessed 8 June 2023.

[61] Arnbjerg CJ, et al. Help-seeking patterns and level of care for individuals with bipolar disorder in Rwanda. PLoS Glob Public Health. 2023;3(10): e0002459. 10.1371/journal.pgph.0002459. (**in English**). 37815957 10.1371/journal.pgph.0002459 PMC10564122

[62] Roberts T, MiguelEsponda G, Torre C, Pillai P, Cohen A, Burgess RA. Reconceptualising the treatment gap for common mental disorders: a fork in the road for global mental health? Br J Psychiatry. 2022;221(3):553–7. 10.1192/bjp.2021.221. (**in English**). 35137680 10.1192/bjp.2021.221

[63] Sorsdahl K, et al. Integration of mental health counselling into chronic disease services at the primary health care level: Formative research on dedicated versus designated strategies in the Western Cape, South Africa. J Health Serv Res Policy. 2021;26(3):172–9. 10.1177/1355819620954232. (**in English**). 32969273 10.1177/1355819620954232 PMC7619420

[64] Rabkin M, El-Sadr WM. Why reinvent the wheel? Leveraging the lessons of HIV scale-up to confront non-communicable diseases. Glob Public Health. 2011;6(3):247–56. 10.1080/17441692.2011.552068. (**in English**). 21390970 10.1080/17441692.2011.552068

[65] Joska JA, Sorsdahl KR. Integrating mental health into general health care: lessons from HIV. Afr J Psychiatry (Johannesbg). 2012;15(6):420–3. 10.4314/ajpsy.v15i6.52. (**in English**). 23160616 10.4314/ajpsy.v15i6.52

[66] Hogg B, et al. Psychological trauma as a transdiagnostic risk factor for mental disorder: an umbrella meta-analysis. Eur Arch Psychiatry Clin Neurosci. 2023;273(2):397–410. 10.1007/s00406-022-01495-5. (**in English**). 36208317 10.1007/s00406-022-01495-5

