# Supplementary material for: Prevalence of Mental Health Disorders and Their Associated Risk Factors Among People Living with HIV in Rwanda: A Cross-Sectional Study
Source: AIDS Behav. 2024 May 12;28(8):2666–82. doi: 10.1007/s10461-024-04358-3 (PMC11286631; doi:10.1007/s10461-024-04358-3)
Supplement: Supplementary file 1 — Supplementary file1 (DOCX 36 KB) [file 10461_2024_4358_MOESM1_ESM.docx]

**Supplementary tables**

Title: Prevalence of mental health disorders and their associated risk factors among People Living with HIV in Rwanda: a cross-sectional study

Journal name: AIDS and Behavior

Author names: Laura Risbjerg Omann, Valentine Dushimiyimana, Emmanuel Musoni-Rwililiza, Caroline Juhl Arnbjerg-Nielsen, Vivianne Umuhire Niyonkuru, Jean Damascene Iyamuremye, Michel Gasana, Jessica Carlsson, Per Kallestrup Christian Kraef

Corresponding author:

Laura Risbjerg Omann

Center for Global Health, Department of Public Health, Aarhus University, Denmark

[lauraomann@hotmail.com](mailto:lauraomann@hotmail.com)

**Table 1a - supplementary**

Table 1a - supplementary. HIV characteristics of participants by diagnostic group.

|  |  | **All**  **(N = 428)** | **No diagnosis (n = 358)** | **Any diagnosis**  **(n = 70)** | **MDE only (n = 46)** | **PTSD only (n = 9)** | **MDE and PTSD**  **(n = 14)** |
| --- | --- | --- | --- | --- | --- | --- | --- |
| **HIV-related data** | | | | | | | |
| **Ever changed ART treatment, n (%)** | Yes | 417 (97.4) | 348 (97.2) | 69 (98.6) | 45 (97.8) | 9 (100.0) | 14 (100.0) |
| **Previous type of ART^p^, n (%)** | TDF/3TC/EFV | 121 (28.8) | 99 (28.2) | 22 (31.9) | 16 (35.6) | 1 (11.1) | 4 (28.6) |
|  | TDF/3TC/DTG | 168 (40.0) | 144 (41.0) | 24 (34.8) | 17 (37.8) | 4 (44.4) | 3 (21.4) |
|  | Other | 120 (28.6) | 98 (27.9) | 22 (31.9) | 11 (24.4) | 4 (44.4) | 7 (50.0) |
|  | Never changed | 11 (2.6) | 10 (2.8) | 1 (1.4) | 1 (2.2) | 0 (0.0) | 0 (0.0) |
| **Reason for ART change^q^, n (%)** | Side effects | 6 (1.4) | 5 (1.4) | 1 (1.4) | 0 (0.0) | 0 (0.0) | 1 (7.1) |
|  | New protocol/guidelines | 370 (86.7) | 313 (87.7) | 57 (81.4) | 37 (80.4) | 7 (77.8) | 12 (85.7) |
|  | Treatment failure | 21 (4.9) | 17 (4.8) | 4 (5.7) | 3 (6.5) | 1 (11.1) | 0 (0.0) |
|  | Toxicity | 7 (1.6) | 6 (1.7) | 1 (1.4) | 0 (0.0) | 0 (0.0) | 1 (7.1) |
|  | Renal toxicity | 9 (2.1) | 5 (1.4) | 4 (5.8) | 3 (6.5) | 1 (11.1) | 0 (0.0) |
|  | Other | 3 (0.7) | 1 (0.3) | 2 (2.9) | 2 (4.3) | 0 (0.0) | 0 (0.0) |
|  | Never changed | 11 (2.6) | 10 (2.8) | 1 (1.4) | 1 (2.2) | 0 (0.0) | 0 (0.0) |
| **Virally suppressed 6 months after ART initiation^r^, n (%)** | Yes | 367 (88.4) | 311 [89.1] | 56 (84.8) | 38 (84.4) | 7 (87.5) | 10 (83.3) |

^p^8 missing

^q^18 missing

^r^13 missing

**Table 2a - supplementary**

Table 2a - supplementary. Risk factors for PTSD and depression and PTSD only compared to no diagnosis.

|  |  | **MDE and PTSD (n = 14)** | | | | **PTSD only (n = 9)** | | | |
| --- | --- | --- | --- | --- | --- | --- | --- | --- | --- |
|  |  | **RR (95% CI)** | **p-value** | **aRR (95% CI)** | **p-value** | **RR (95% CI)** | **p-value** | **aRR (95% CI)** | **p-value** |
| **Sociodemographic data** | | | | | | | | | |
| **Age group** | <=24 | Ref | Ref |  |  | Ref | Ref |  |  |
|  | 25-34 |  |  |  |  |  |  |  |  |
|  | 35-44 | 0.86 (0.11-6.99) | 1.00 | 1.56 (0.28-8.67) | 0.61 |  |  |  |  |
|  | 45-54 | 0.54 (0.06-4.95) | 0.49 | 1.05 (0.16-7.01) | 0.96 |  |  |  |  |
|  | >=55 | 1.27 (0.16-10.2) | 1.00 | 1.91 (0.38-9.56) | 0.43 |  |  |  |  |
| **Gender** | Female | 2.29 (0.65-8.07) | 0.26 | 2.02 (0.62-6.60) | 0.25 | 4.99 (0.63-39.48) | 0.16 | 5.67 (1.42-22.64) | 0.014 |
| **Civil status** | Single | Ref | Ref |  |  | Ref | Ref |  |  |
|  | Married or cohabitant | 1.59 (0.20-12.96) | 1.00 |  |  | 0.55 (0.10-2.90) | 0.61 |  |  |
|  | Divorced or separated | 3.73 (0.35-39.51) | 0.28 |  |  | 1.90 (0.28-12.8) | 0.61 |  |  |
|  | Widower | 3.78 (0.45-31.48) | 0.24 |  |  | 0.41 (0.04-4.38) | 0.59 |  |  |
| **Educational level** | No formal schooling or less than primary school | Ref | Ref |  |  | Ref | Ref |  |  |
|  | Primary school completed | 1.23 (0.42-3.59) | 0.79 |  |  | 1.37 (0.35-5.37) | 0.74 |  |  |
|  | High school or above completed |  |  |  |  |  |  |  |  |
| **Occupation** | Other (including government and non-government employee, student, other) | Ref | Ref |  |  | Ref | Ref |  |  |
|  | Self-employed | 0.79 (0.11-5.49) | 1.00 |  |  |  |  |  |  |
|  | Unemployed (including volunteer, homemaker, or retired) | 0.53 (0.05-5.70) | 1.00 |  |  |  |  |  |  |
|  | Farmer | 2.71 (0.60-12.2) | 0.21 |  |  |  |  |  |  |
| **Socioeconomic class (Ubudehe)** | 1 | Ref | Ref | Ref | Ref | Ref | Ref |  |  |
|  | 2 | 0.27 (0.06-1.17) | 0.097 | 0.54 (0.11-2.66) | 0.45 | 1.14 (0.14-9.18) | 1.00 | 2.24 (0.31-16.06) | 0.42 |
|  | 3 or 4 | 0.47 (0.13-1.74) | 0.38 | 1.41 (0.28-7.11) | 0.68 | 0.39 (0.04-4.20) | 0.42 | 1.18 (0.12-11.93) | 0.89 |
| **DSDM category** | Stable A1 | Ref | Ref | Ref | Ref | Ref | Ref | Ref | Ref |
|  | Stable A2 | 3.77 (1.15-12.35) | 0.043 | 2.60 (0.69-9.77) | 0.16 | 2.75 (0.55-13.72) | 0.22 | 1.62 (0.22-11.87) | 0.63 |
|  | Stable B |  |  |  |  | 8.68 (1.86-40.58) | 0.033 | 9.95 (2.19-45.2) | 0.0029 |
|  | Unstable | 3.58 (0.97-13.20) | 0.079 | 3.39 (0.94-12.19) | 0.062 |  |  |  |  |
| **HIV-related data** | | | | | | | | | |
| **Type of ART** | TDF/3TC/DTG | Ref | Ref |  |  | Ref | Ref |  |  |
|  | TDF/3TC/EFV | 1.03 (0.31-3.42) | 1 |  |  | 1.20 (0.20-7.03) | 1.00 |  |  |
|  | Other | 0.80 (0.21-3.03) | 1 |  |  | 2.42 (0.55-10.6) | 0.25 |  |  |
| **Virally suppressed at last visit** | Yes (vs no) | 0.40 (0.057-2.79) | 0.35 |  |  |  |  |  |  |
| **Virally suppressed at visit 6 months after ART initiation** | Yes (vs no) | 0.62 (0.14-2.74) | 0.63 |  |  | 0.86 (0.11-6.79) | 1 |  |  |
| **Psychosocial data** | | | | | | | | | |
| **Has person(s) to trust** | No (vs yes) |  |  |  |  | 1.71 (0.22-13.14) | 0.47 |  |  |
| **Family history of mental illness** | Yes (vs no) | 2.90 (0.85-9.86) | 0.11 | 2.50 (0.71-8.82) | 0.15 |  |  |  |  |
| **Self-reported symptoms/diagnosis of mental health disorders before HIV diagnosis** | Yes (vs no) | 5.6 (0.90-35.00) | 0.18 | 4.74 (0.53-42.37) | 0.16 | 8.98 (1.37-58.95) | 0.12 | 25.87 (1.40-479.64) | 0.029 |
| **Self-reported symptoms/diagnosis of mental health disorders after HIV diagnosis** | Yes (vs no) | 11.93 (4.36-32.66) | 0.00054 |  |  | 4.94 (0.69-35.48) | 0.20 |  |  |
| **Currently or previously in treatment for mental health problems** | Yes (vs no) |  |  |  |  |  |  |  |  |
| **Negative feelings related to HIV status** | Yes (vs no) | 8.20 (3.04-22.13) | 0.00039 |  |  | 6.02 (1.59-22.78) | 0.025 |  |  |
| **HIV status has impacted the ability to engage in social relationships negatively** | Yes (vs no) | 1.35 (0.39-4.72) | 0.71 | 0.73 (0.19-2.79) | 0.65 | 1.42 (0.30-6.69) | 0.65 | 0.27 (0.05-1.43) | 0.12 |
| **HIV status has impacted sexual life negatively** | Yes (vs no) | 1.59 (0.37-6.80) | 0.63 | 1.58 (0.41-6.08) | 0.51 | 1.21 (0.16-9.38) | 0.59 | 0.86 (0.11-6.43) | 0.88 |
| **Experiences of HIV stigma/discrimination** | Yes (vs no) | 2.70 (0.94-7.78) | 0.071 | 1.94 (0.63-6.01) | 0.25 | 5.97 (1.65-21.62) | 0.0096 | 10.51 (2.32-47.60) | 0.0023 |
| **If yes to above: HIV stigma and discrimination have affected mental well-being negatively** | Yes (vs no) | 6.5 (0.77-54.78) | 0.065 |  |  | 2.61 (0.47-14.48) | 0.35 |  |  |

Note: Multivariable model is adjusted for age group, gender, socioeconomic class, DSDM category, family history of mental illness, self-reported mental health symptoms or diagnosis, if HIV status had negative impact on social relations or sexual life, and HIV-related stigma and discrimination.

For the variables with missing RR and aRR, 95% confidence intervals, and p-values, these were not reported as they were not possible to calculate due to the small number of participants in each group.

**Table 3 - supplementary**

Table 3 - supplementary. Study participants by site (October 2022).

| **Health facility** | **CHK(CHUK) University Teaching Hospital of Kigali** | **Cor-unum HC^s^** | **Kabuga (kicukiro) HC** | **Kinyinya HC** | **Gisenyi HC** | **Gisenyi DH^t^** | **Kabgayi HC** | **Kabgayi DH** | **Muhoza (Ruhengeri) HC** | **Ruhengeri RH^u^** | **Rwamagana HC** | **Rwamagana PH^v^** |
| --- | --- | --- | --- | --- | --- | --- | --- | --- | --- | --- | --- | --- |
| **Province** | Kigali City | Kigali City | Kigali City | Kigali City | West | West | South | South | North | North | East | East |
| **District** | Nyarugenge District | Nyarugenge District | Kicukiro District | Gasabo District | Rubavu District | Rubavu District | Muhanga District | Muhanga District | Musanze District | Musanze District | Rwamagana District | Rwamagana District |
| **Geographical location** | Urban | Urban | Urban | Urban | Rural | Rural | Rural | Rural | Rural | Rural | Rural | Rural |
| **Number of PLWH at the site** | 2095 | 2885 | 913 | 2312 | 781 | 1308 | 792 | 1380 | 1045 | 1427 | 834 | 907 |
| **%Male PLWH** | 41.0 | 37.0 | 38.0 | 35.0 | 28.0 | 40.7 | 29.0 | 43.0 | 24.0 | 37.0 | 31.0 | 41.0 |
| **%Age male PLWH** |  | | | | | | | | | | | |
| **≤ 24** | 9 | 2 | 4 | 4 | 4 | 9 | 1 | 9 | 1 | 10 | 2 | 7 |
| **25-34** | 10 | 17 | 16 | 13 | 31 | 10 | 22 | 6 | 26 | 9 | 21 | 8 |
| **35-44** | 22 | 45 | 36 | 33 | 40 | 22 | 46 | 19 | 38 | 18 | 38 | 17 |
| **45 -54** | 30 | 25 | 28 | 28 | 15 | 30 | 19 | 34 | 22 | 35 | 26 | 36 |
| **≥ 55** | 29 | 11 | 15 | 22 | 9 | 29 | 12 | 32 | 13 | 28 | 13 | 32 |
| **%Female PLWH** | 59.0 | 63.0 | 62.0 | 65.0 | 72.0 | 59.3 | 71.0 | 57.0 | 76.0 | 63.0 | 69.0 | 59.0 |
| **%Age female PLWH** |  | | | | | | | | | | | |
| **≤ 24** | 4 | 6 | 6 | 7 | 7 | 4 | 10 | 4 | 7 | 6 | 5 | 7 |
| **25-34** | 6 | 30 | 29 | 24 | 41 | 6 | 32 | 5 | 38 | 6 | 37 | 7 |
| **35-44** | 27 | 35 | 36 | 30 | 42 | 27 | 36 | 17 | 39 | 21 | 36 | 14 |
| **45 -54** | 41 | 20 | 19 | 27 | 7 | 41 | 15 | 45 | 13 | 40 | 16 | 41 |
| **≥ 55** | 22 | 9 | 9 | 12 | 3 | 22 | 7 | 29 | 4 | 27 | 6 | 31 |
| **Number of PLWH needed to be included from site** | 55 | 76 | 24 | 59 | 21 | 34 | 21 | 36 | 28 | 37 | 22 | 24 |

^s^HC: Health Facility

^t^DH: District Hospital

^u^RH: Referral Hospital

^v^PH: Province Hospital
